# Memorial Resolution

**Published:** 1915-11

**Authors:** 


### MEMORIAL RESOLUTION.

### Adopted by the Faculty of the Northwestern University Dental School on Thursday Evening, September 23, 1915, at a Special Meeting Convened to Commemorate the Life and Service of Dean Greene Vardiman Black.

---

The following resolution was prepared and presented by Doctors Noyes and Gilmer and was unanimously adopted by a rising vote:

The faculty and teaching staff of Northwestern University Dental School enter upon their records this memorial of their beloved dean, Greene Vardiman Black, M.D., D.D.S., Sc.D., LL.D., who died August 31, 1915, at the age of seventy-nine years.

Dr. Black was a teacher in dental schools for forty-five years, with an interruption of only three years, during which time he was the first president of the Illinois State Board of Dental Examiners, in which office he continued for four years more after he resumed teaching (1881 to 1887). His first school work was as lecturer on pathology, histology and operative dentistry in the Missouri Dental College, 1870-1880. He was professor of dental pathology in the Chicago College of Dental Surgery, 1883-1889. During this time, in 1887, he introduced the teaching of dental technics, by which the teaching of technical procedures in both operative and prosthetic dentistry are taught in laboratories, instead of depending, as previously, upon practical work in the clinic to acquire knowledge and skill in technical operations. This was an entirely new departure in dental teaching which was very soon adopted by most of the schools throughout the country, and finally by all of them. He was professor of dental pathology and bacteriology in the dental department of the University of Iowa, 1890-1891. In the latter year (1891) he entered the faculty of this school as professor of dental pathology and bacteriology till 1897, when he became dean and professor of operative dentistry, dental pathology and bacteriology, and continued until his death.

The most important of Dr. Black's books are the chapters he wrote for the "American System of Dentistry," "The Periosteum and Peridental Membrane," the "Dental Anatomy," the "Operative Dentistry" and the "Special Dental Pathology."

His nature was simple, sincere and approachable, and everyone who showed an interest in dental matters received a cordial welcome and always found him ready to impart knowledge. He was a friendly man and attracted everyone who came within the sphere of his influence. The greatest desire and ambition of his life was to improve the standards and methods of dental education.

While in practice he was a very exceptionally wise and skillful operator, and throughout his long life he was a hard student, a successful investigator and inventor and probably the most useful and the most influential man in the dental profession.

It would be interesting to tell of Dr. Black's studies and investigations that did not relate to dentistry. Two may be mentioned. He at one time made a study of the rings of annual growth in tree stumps, to find out which were the wet and which the dry seasons, recorded in the varying thickness of the annual growth, and he found that his interpretations corresponded accurately with the recorded weather reports as far back as there were any such reports. At another time he worked out the life history of thirty or forty varieties of households. He was a many-sided man, and could do an astonishing number of different things, and do them better than other men could do them.

The honor, admiration and affection we all felt for him can be only feebly expressed.

We also desire to express to Mrs. Black, the devoted wife, our most sincere and deep-felt sympathy in her bereavement. While making full recognition of the services of her distinguished husband, we wish to express our belief that she was no small factor in his great achievements, and we desire to acknowledge at this time the sacrifices she made in aid of the success of his labors.

